# Mental well-being and job satisfaction among general practitioners: a nationwide cross-sectional survey in Denmark

**DOI:** 10.1186/s12875-018-0809-3

**Published:** 2018-07-28

**Authors:** Karen Busk Nørøxe, Anette Fischer Pedersen, Flemming Bro, Peter Vedsted

**Affiliations:** 1 Research Unit for General Practice, Department of Public Health, Aarhus University, Bartholins Allé 2, 8000 Aarhus C, Denmark; 2 Research Unit for General Practice & Department of Clinical Medicine, Aarhus University, Aarhus, Denmark

**Keywords:** General practitioner, Primary care, Burnout, Job satisfaction, Mental health, Work-life balance, Denmark

## Abstract

**Background:**

Poor mental well-being and low job satisfaction among physicians can have significant negative implications for the physicians and their patients and may also reduce the cost efficiency in health care. Mental distress is increasingly common in physicians, including general practitioners (GPs). This study aimed to examine mental well-being and job satisfaction among Danish GPs and potential associations with age, gender and practice organisation.

**Methods:**

Data was collected in a nationwide questionnaire survey among Danish GPs in 2016. Register data on GPs and their patient populations was used to explore differences between respondents and non-respondents. Associations were estimated using multivariate logistic regression analysis.

**Results:**

Of 3350 eligible GPs, 1697 (50.7%) responded. Lower response rate was associated with increasing numbers of comorbid, aging or deprived patients. About half of participating GPs presented with at least one burnout symptom; 30.6% had high emotional exhaustion, 21.0% high depersonalisation and 36.6% low personal accomplishment. About a quarter (26.2%) experienced more than one of these symptoms, and 10.4% experienced all of them. Poor work-life balance was reported by 16.2%, low job satisfaction by 22.1%, high perceived stress by 20.6% and poor general well-being by 18.6%. Constructs were overlapping; 8.4% had poor overall mental health, which was characterized by poor general well-being, high stress and ≥ 2 burnout symptoms. In contrast, 24.6% had no burnout symptoms and reported high levels of general well-being and job satisfaction. Male GPs more often than female GPs reported low job satisfaction, depersonalisation, complete burnout and poor overall mental health. Middle-aged (46–59 years) GPs had higher risk of low job satisfaction, burnout and suboptimal self-rated health than GPs in other age groups. GPs in solo practices more often assessed the work-life balance as poor than GPs in group practices.

**Conclusion:**

The prevalence of poor mental well-being and low job satisfaction was generally high, particularly among mid-career GPs and male GPs. Approximately 8% was substantially distressed, and approximately 25% reported positive mental well-being and job satisfaction, which shows huge variation in the mental well-being among Danish GPs. The results call for targeted interventions to improve mental well-being and job satisfaction among GPs.

**Electronic supplementary material:**

The online version of this article (10.1186/s12875-018-0809-3) contains supplementary material, which is available to authorized users.

## Background

Mental distress such as perceived stress and burnout is common in physicians. It seems to be an escalating problem, also among general practitioners (GPs) [1–7]. The negative implications of dissatisfaction and mental distress are far-reaching for the affected physicians, but they may also influence the quality of patient care and the cost-efficiency in health care [8–10].

Physicians experience continuous changes in their working conditions. These are caused by the changing needs and demands of the population, expansion of the medical knowledge, new health technology and reorganisation of the healthcare systems. The transfer of medical care from secondary to primary care, more administrative tasks, an aging population and the growing prevalence of people with chronic conditions have increased the workload in primary care. Together with workforce concerns, these changes may have affected the mental well-being and the job satisfaction among GPs [11–13].

The positive aspects of the clinical work among GPs are generally related to the close relationship with the patients and the provision of high-quality and continuous patient care [13–16]. However, such positive aspects may be eroded by time constraints and organizational changes aiming to cope with increasing workloads [17–19].

The past decade has seen a rise in the clinical workload in primary care when measured as number of patient contacts per GP [20, 21]. A further increase in the clinical workload is expected as the elderly population continues to grow, and more people live with chronic disease. Patients with multiple diagnoses often consult their GP and have several contacts to specialised care; these consultations are often complex, time-consuming and perceived as demanding by GPs [22–24]. In line with this, caring for a practice population with a high share of deprived patients has been shown to be associated with GP burnout [25].

Burnout is considered a prolonged response to work-related distress that evolves gradually over time. It has been described as an erosion of engagement; energy turns into exhaustion, involvement into cynicism and efficacy into perceived ineffectiveness [26, 27].

Motivated by concerns of increasing prevalence of mental distress among Danish GPs and potential implications thereof, we conducted a nationwide study aiming to explore changes in mental well-being and job satisfaction among Danish GPs and potential associations with age, gender and practice organisation.

## Methods

### Setting

Danish GPs work as independent contractors for the regional health authorities. GPs working under this tax-financed public reimbursement system are organised in the Organisation of General Practitioners in Denmark (PLO).

Almost all citizens (99%) are listed with a specific general practice, which they must consult (free of charge) for medical advice. Danish GPs act as gatekeepers to the rest of the health care system (except for emergencies), and the GPs provide comprehensive primary care (including preventive maternal and child care) with high levels of continuity [28]. Listed patients on average consult the general practice nearly seven times annually, and the average list size is approximately 1600 patients per GP [21]. The GPs must provide medical care all weekdays from 8 am to 4 pm, and all acute situations must be dealt with on the same day. Many GPs are also obligated to participate in out-of-hours cooperatives.

### Study population

**Fig. 1:**
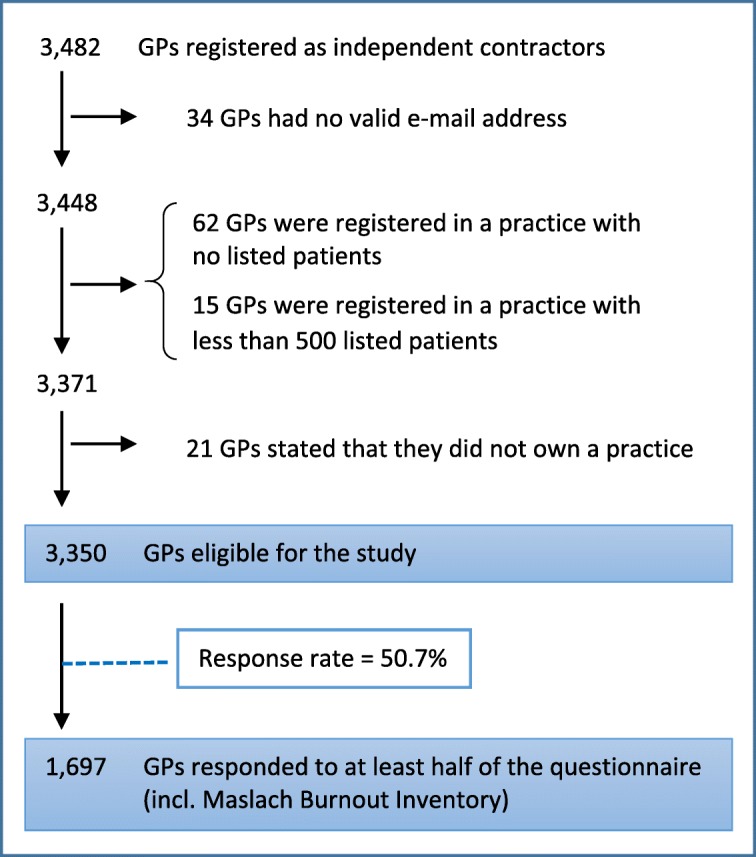
Flowchart of GPs included in the study

We included only GPs who were independent contractors (owners) working with the regional health authorities (excluding locums and trainees) in practices with at least 500 listed patients. A total of 3350 GPs were eligible for inclusion (Fig. 1).

### Data collection

All Danish GPs listed with a valid email address at PLO in May 2016 received an email with a link to an electronically administered questionnaire. The survey was announced 1 week before by email and in a newsletter on the PLO website. Non-respondents received a reminder after 2 weeks and after 4 weeks, and the data collection terminated on 1 July 2016. The link to the questionnaire was personal; it contained a unique serial number but no personal identifiers. The PLO distributed the link, and the research group collected the survey data. The PLO provided administrative data on the GPs. The data was transferred to Statistics Denmark separately; survey data by the research group and administrative data by the PLO. The data was linked at Statistics Denmark by the unique serial number, which was subsequently deleted and combined with register data by encrypted identifiers for anonymous analysis.

### Questionnaire survey

The questionnaire was developed from themes identified in existing literature and interviews with experienced researchers and clinicians. The interviews included eight individual telephone interviews (with seven GPs and one social worker employed by the Danish Medical Association) and a focus-group interview involving a representative from the Collegial Network for Physicians in Denmark (an organisation offering support to physicians), an occupational psychologist employed by the Danish Medical Association and a GP.

We used validated scales whenever possible and constructed ad hoc items when validated scales were unavailable. Scales and items were discussed and agreed upon in the research group. A pilot test was conducted among 10 GPs to assess the relevance and comprehensiveness of the questionnaire battery. Only minor changes (deletion of a few ad hoc items) were made based on the pilot test.

The following scales were used in this study: the Maslach Burnout Inventory-Human Services Survey (MBI-HSS), the 10-item Danish version of Cohen’s Perceived Stress Scale (PSS-10), the Warr-Cook-Wall Job Satisfaction Scale (WCW-JSS) and the World Health Organisation (Five) Well-Being Index (WHO-5). In addition, items concerning self-rated health (from the 12-item Short Form Health Survey (SF-12)), strains in private life and work-life balance were used.

Included scales have previously been translated for research use by standardised procedures; these generally include a forward translation carried out by researchers and a linguistic expert, backward translation by a native English-speaking person who was fluent in Danish, panel discussion and pilot test [29, 30].

Additional items concerned topics such as GP demographics, practice organisation, working hours, potential job-related strains, stress management, presentism and self-rated quality of clinical work. A free-text field was also added at the end of the questionnaire.

The GPs were required to respond to all items in the specific scale in order to proceed; this setup ensured that no items were missing. Completing the questionnaire took approximately 25 min.

### Other data

The administrative data on the GPs included civil registration number (CRN), age, gender, region and provider number. The number of GPs registered with each practice was categorised into: 1 GP, 2–3 GPs and >  3 GPs. Practices with one GP were defined as solo practices and practices with two or more GPs as group practices. GP age was categorised into three age groups: ≤ 45, 46–59 and ≥ 60 years.

Data on patients listed with each practice was collected from national registers. All Danish citizens are assigned a unique CRN, which allows accurate linkage of information from numerous different registers at the individual level [31].

The number of listed patients per GP was calculated as practice list size divided by the number of GPs. This was done for all listed patients, patients ≥70 years and patients with a score of ≥1 in Charlson’s Comorbidity Index (CCI). The CCI was computed based on all primary and secondary diagnoses in the Danish National Patient Register (both inpatient and outpatient hospital diagnoses) from 2006 to 2016 [32, 33].

The socio-economic burden within the practice population was measured by the Danish Deprivation Index (DADI) [25], which estimates the socio-economic burden based on eight key variables. Higher values indicate a higher proportion of deprived patients. The number of patients per GP (all patients and subgroups) and DADI scores were categorised into quartiles based on all eligible GPs and practices. Information on the practice population was collected at the end of 2015.

### Outcome measures

**Fig. 2:**
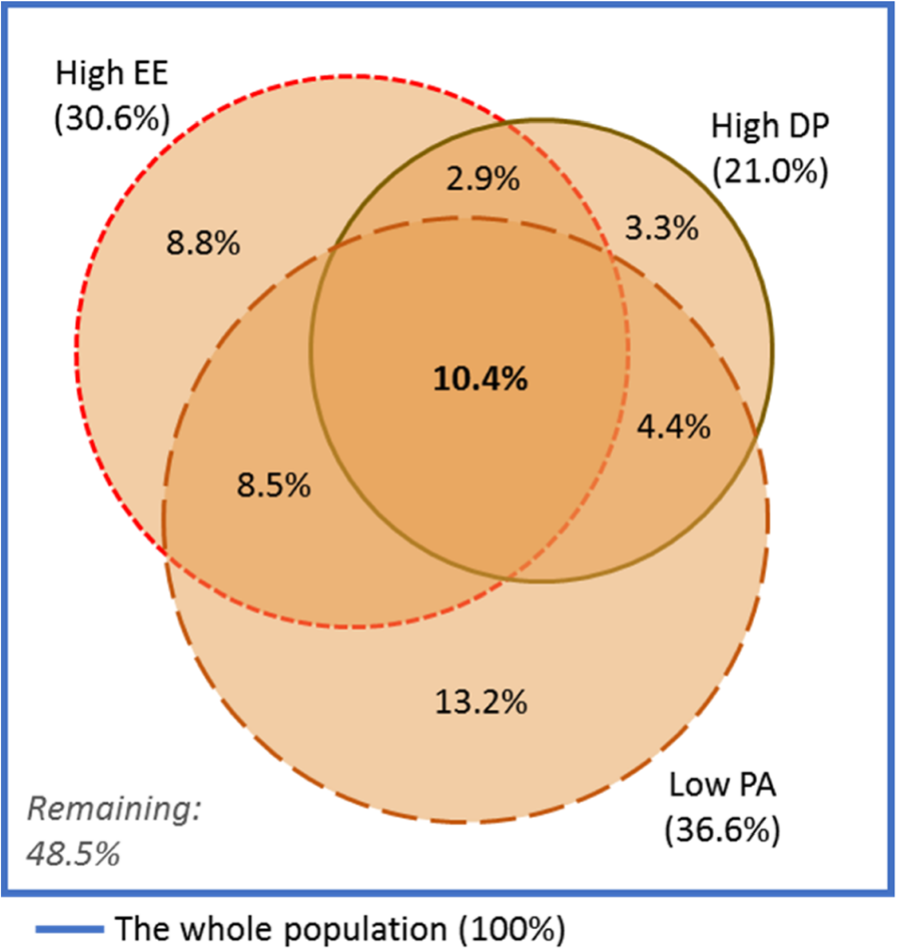
Percentage of GPs reporting high emotional exhaustion (EE), high depersonalisation (DP) and low personal accomplishment (PA). (*N* = 1697 GPs who responded to all MBI-HSS subscales)

**Burnout and engagement** was measured by the MBI-HSS. This instrument is considered a gold standard for assessment of burnout. It measures three burnout dimensions: (1) emotional exhaustion (EE), which is characterised by depletion of emotional resources, (2) depersonalisation (DP), which is characterised by emotional detachment from people related to work including patients, and (3) personal accomplishment (PA), which includes perceived value of work and self-efficacy. Subscale sum scores reflect the degree of burnout on each dimension. Based on predefined cut-off values for healthcare workers, each subscale score was defined as low, moderate or high [34]. Overlap between subscale scores that were indicative of burnout (high EE, high DP and low PA) was reported (Fig. 2). Complete burnout syndrome was defined as burnout-indicative scores on all MBI subscales. The opposite positive pole (low EE, low DP and high PA) was labelled ‘engagement’ [26, 27].

**Job satisfaction** was measured by the WCW-JSS. Nine job satisfaction facets and the overall job satisfaction were rated on a scale from 1 (‘extreme dissatisfaction’) to 7 (‘extreme satisfaction’) and added up to a sum score [35]. As no pre-determined cut-off values exist, respondents were divided into quartiles based on their sum score. For descriptive statistics, the GPs’ own ratings of their overall job satisfaction (single item) were used: a score of ≤3 was categorised as low, 4–5 as moderate and ≥ 6 as high job satisfaction. The single item had high correlation with the full scale (Pearson’s correlation coefficient: 0.89).

**Perceived stress** was measured by the PSS-10 [36, 37]. This widely used instrument consists of ten items about the frequency of stress-related feelings and thoughts. Each item is rated on a scale from 0 (‘never’) to 4 (‘very often’). In accordance with previous research, a sum score of ≥18 was considered as high level of perceived stress [38].

**General well-being** was measured by the WHO-5, which consists of five positively phrased items about cheerfulness, calmness, energy and interest in day-to-day activities in the previous 14 days. Items are added up and multiplied by four to create a scale from 0 (worst quality of life possible) to 100 (best quality of life possible). In general populations, the mean WHO-5 score is 70. When screening for depression, a cut-off score of ≤50 is recommended [45]. We divided general well-being into three categories: ‘high’ for a score of > 70, ‘poor’ for a score of ≤50 and ‘moderate’ for a score in between.

**Self-rated health** was assessed by a single item from the SF-12 asking respondents to rate their general health as ‘excellent’, ‘very good’, ‘good’, ‘fair’ or ‘poor’ [39].

**Work-life balance** was assessed by one item: *‘Do you generally experience a good balance between work and private life?*’ Responses were categorised as ‘good’ (‘*to a very high degree’* and *‘to a high degree’), ‘*moderate’ (‘*partly*’) or ‘poor’ (*‘to a low degree’* and *‘to a very low degree’).*

**Strains in private life** were assessed by one item: ‘*Do you feel burdened by factors related to your private life (economic issues, family problems, health conditions or similar)?’* Responses were categorised as ‘*No*’, ‘*Yes, but it burdens me only little*’, ‘*Yes, and it burdens me to some extent*’, ‘*Yes, and it burdens me a lot*’ and ‘*I do not know/I do not wish to answer*’. The latter response category was classified as ‘missing’.

### Analysis

GPs who completed at least half of the questionnaire (including the WCW-JSS and the MBI-HSS) were classified as respondents. The response rate within subgroups and the corresponding 95% confidence interval (CI) were calculated. The response rate within subgroups was further adjusted for gender, age, GPs per practice, listed patients per GP and DADI score, and these estimates were presented as risk difference (RDs) with 95% CI.

For each scale, we computed mean sum score, standard deviation (SD), median sum score and interquartile interval (IQI). Within each category of well-being and satisfaction, the percentage of GPs was calculated with 95% CI.

Overlap of burnout symptoms (high EE, high DP and low PA) was visualised in an area-proportional Venn diagram. Likewise, we made Venn diagrams of negative mental health aspects (≥ 2 burnout symptoms, high perceived stress and poor general well-being) and of positive mental health aspects (no symptoms of burnout and high general well-being) and job satisfaction.

Associations between outcome measures and selected GP characteristics (age, gender and type of practice) were estimated as odds ratios (OR) with 95% CI using logistic regression while adjusting for the mentioned GP characteristics.

Scale performance was assessed by Cronbach’s alpha, average inter-item correlation, and floor and ceiling effects. Analyses were performed in Stata, version 12. *P*-values of ≤0.05 were considered statistically significant.

## Results

Out of 3350 eligible GPs, 1697 (50.7%) responded (Fig. 1). The response rate varied according to GP characteristics (Table 1). In the adjusted analyses, the response rate was lower among men than among women and lower among GPs aged ≥60 years than among GPs aged ≤45 years. Increasing number of elderly patients per GP, number of patients with morbidity per GP and higher deprivation index were all factors associated with lower response rate (Table 1).

**Table 1:** Characteristics of eligible GPs and survey respondents

		Eligible GPs	Respondents	Response rate	Risk difference, adjusted^a^
		N (%)	N (%)	% (95% CI)	PP^b^ (95% CI)
Total		3350 (100)	1697 (100)	50.7 (48.9–52.4)	
Gender	Female	1670 (49.9)	941 (55.5)	56.3 (53.9–58.7)	ref.
	Male	1680 (50.1)	756 (44.5)	45.0 (42.6–47.4)	**-8.2 (-11.7; -4.7)**
Age (years)	≤ 45	934 (27.9)	501 (29.5)	53.6 (50.4–56.9)	ref.
	46–59	1404 (41.9)	771 (45.4)	54.9 (52.2–57.5)	1.7 (-2.4–5.9)
	≥ 60	1012 (30.2)	425 (25.0)	42.0 (38.9–45.1)	**-8.0 (-12.7; -3.3)**
GPs per practice	1	965 (28.8)	439 (25.9)	45.5 (42.3–48.7)	ref.
	2–3	1515 (45.2)	786 (46.3)	51.9 (49.4–54.5)	1.3 (-3.0–5.6)
	> 3	871 (26.0)	472 (27.8)	54.2 (50.8–57.5)	2.4 (-2.6–7.3)
Listed patients per GP (number):					
All patients	< 1400	841 (25.1)	442 (26.1)	52.6 (49.1–56.0)	ref.
	1400–1589	836 (25.0)	456 (26.9)	54.5 (51.1–58.0)	2.1 (-2.7–6.8)
	1590–1779	836 (25.0)	416 (24.5)	49.8 (46.3–53.2)	− 2.4 (-7.2–2.4)
	> 1779	837 (25.0)	383 (22.6)	45.8 (42.3–49.2)	**-5.4 (-10.3; -0.5)**
Patients with CCI score of ≥1	< 224	846 (25.3)	471 (27.8)	55.7 (52.3–59.1)	ref.
	224–263	837 (25.0)	446 (26.3)	53.3 (49.8–56.7)	-2.9 (-7.9–2.0)
	264–311	836 (25.0)	416 (24.5)	49.8 (46.3–53.2)	**-5.0 (-10.3; -0.5)**
	> 311	831 (24.8)	364 (21.5)	43.8 (40.4–47.3)	**-7.6 (-13.5; -1.6)**
Patients aged ≥70 years	< 152	839 (25.0)	468 (27.6)	55.8 (52.3–59.2)	ref.
	152–200	840 (25.1)	433 (25.5)	51.5 (48.1–55.0)	**-4.8 (-9.6–0.0)**
	201–250	839 (25.0)	426 (25.1)	50.8 (47.3–54.2)	**-5.2 (-10.2; -0.3)**
	> 250	832 (24.8)	370 (21.8)	44.4 (41.1–47.9)	**-7.0 (-12.2; -1.8)**
DADI^c^ score	≤ 22.75	842 (25.2)	469 (27.7)	55.7 (52.3–59.1)	ref.
(missing information: *n* = 6)	23–27.25	837 (25.0)	439 (25.9)	52.5 (49.0–55.9)	-2.6 (-7.4–2.1)
	27.5–31.75	834 (24.9)	404 (23.8)	48.9 (45.0–51.9)	**-5.5 (-10.3; -0.8)**
	≥ 32	831 (24.9)	381 (22.5)	45.8 (42.4–49.3)	**-6.4 (-11.3; -1.5)**

^a^Adjusted for gender, age, GPs per practice, number of listed patients per GP and DADI score in categories as presented in the table

^b^*PP* percentage points

^c^*DADI* Danish deprivation index

**Bold** indicates statistically significant difference in adjusted response rate (*p* ≤ 0.05)

The internal consistency of all included scales was adequate, with Cronbach’s alpha coefficient ranging from 0.78 to 0.92. The mean sum scores of the WCW-JSS and the WHO-5 were 48.3 (SD: 13.2) and 65.7 (SD: 18.2), respectively (see the table in the Additional file 1).

Table 2 shows the prevalence of positive and negative mental health and of job satisfaction. When we combined high EE (30.6%), high DP (21.0%) and low PA (36.6%), 26.2% of GPs had high burnout scores on at least two subscales, and 10.4% experienced the complete burnout syndrome. A total of 48.5% reported no symptoms of burnout (Fig. 2). Low job satisfaction was reported by 22.1% of participants, high stress by 20.6% and poor general well-being by 18.6%.

**Table 2:** Reported well-being and job satisfaction among participating GPs

	*n*	% (95% CI)
Burnout/engagement (MBI)		
Emotional exhaustion (EE)		
High (indicative of burnout)	519	30.6 (28.4–32.8)
Medium	501	29.5 (27.4–31.8)
Low	677	39.9 (37.6–42.3)
Depersonalisation (DP)		
High (indicative of burnout)	357	21.0 (19.1–23.1)
Medium	505	29.8 (27.6–32.0)
Low	835	49.2 (46.8–51.6)
Personal accomplishment (PA)		
Low (indicative of burnout)	621	36.6 (34.3–38.9)
Medium	810	47.7 (45.3–50.1)
High	266	15.7 (13.9–17.5)
Overall job satisfaction (WCW-JSS, single item)		
Low (score ≤ 3)	375	22.1 (20.1–24.1)
Moderate (score 4–5)	564	33.2 (31.0–35.5)
High (score ≥ 6)	758	44.7 (42.3–47.1)
Perceived stress (PSS-10)		
Score ≥ 18 (indicative of high stress)	345	20.6 (18.7–22.6)
General well-being (WHO-5)		
Poor (score ≤ 50)	312	18.6 (16.8–20.6)
Moderate	506	30.3 (28.1–32.5)
Good (score > 70)	855	51.1 (48.7–53.5)
Self-rated health (SF-12, single item)		
Poor	6	0.4 (0.1–0.8)
Fair	129	7.7 (6.5–9.1)
Good	545	32.6 (30.3–34.9)
Very good or excellent	994	59.4 (57.0–61.7)
Work-life balance		
Poor	272	16.2 (14.5–18.1)
Moderate	743	44.4 (42.0–46.8)
Good	660	39.4 (37.1–41.8)
Strains in private life		
No	935	56.6 (54.2–59.0)
A little	419	25.4 (23.3–27.5)
Some	250	15.1 (13.4–17.0)
A lot	48	2.9 (2.1–3.8)

**Fig. 3:**
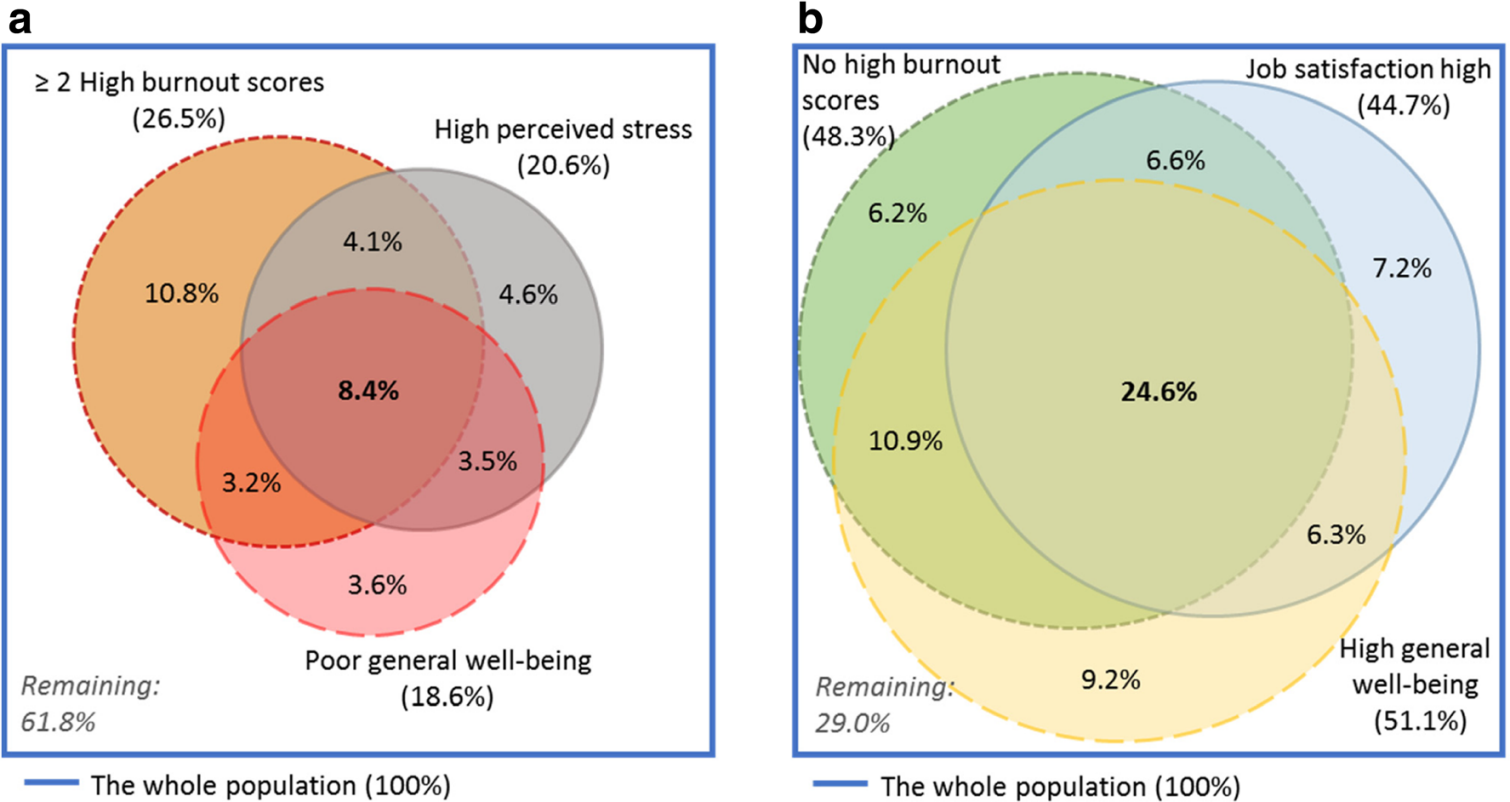
**a** Percentage of GPs with burnout indicative scores on at least 2 subscales, high perceived stress (PSS-10 ≥ 18) and poor general well-being (WHO-5 ≤ 50). (*N* = 1673 GPs who responded to all items). **b** Percentage of GPs without any burnout indicative subscale scores, high overall job satisfaction (single item score ≥ 6) and high general well-being (WHO-5 > 70). (N = 1673 who GPs responded to all items)

Constructs were overlapping; 8.4% had poor overall mental health characterised by poor general well-being, high stress and substantial burnout (Fig. 3a), while 24.6% experienced positive mental health (high general well-being and no symptoms of burnout) and high job satisfaction (Fig. 3b).

The work-life balance was reported as good by 39.4% and poor by 16.2% (Table 2). Strains related to private life burdened 2.9% to a high degree. The general health was rated as just fair by 7.7% and outright poor by 0.4%.

Table 3 shows adjusted associations between mental well-being and GP characteristics. Male GPs were more likely than female GPs to experience low job satisfaction (OR = 1.55 (95% CI: 1.23–1.95)) and poor mental well-being, including complete burnout syndrome (OR = 1.50 (95% CI: 1.08–2.07)) and poor overall mental health (OR = 1.65 (95% CI: 1.16–2.36)). In contrast, male GPs were more likely to experience a good work-life balance (OR = 1.31 (95% CI: 1.07–1.62)).

**Table 3:** Associations between GP-reported well-being and job satisfaction and gender, age and type of practice. Odds ratios with 95% confidence intervals adjusted for GP gender, age and type of practice

	Male	46–59 years	≥ 60 years	Group practice
	(female as ref.)	(≤ 45 years as ref.)		(solo practice as ref.)
**Negative aspects**				
EE high	1.00 (0.81–1.24)	1.15 (0.90–1.46)	**0.70 (0.51–0.94)**	0.84 (0.65–1.07)
DP high	**1.56 (1.23–1.99)**	0.96 (0.73–1.26)	**0.50 (0.35–0.71)**	0.92 (0.70–1.22)
PA low	1.11 (0.91–1.37)	1.15 (0.91–1.46)	0.96 (0.72–1.28)	0.85 (0.68–1.07)
Job satisfaction low^a^	**1.55 (1.23–1.95)**	**1.44 (1.10–1.90)**	1.32 (0.96–1.86)	0.93 (0.72–1.20)
Perceived stress high	0.83 (0.65–1.08)	1.06 (0.81–1.40)	**0.49 (0.34–0.71)**	0.88 (0.66–1.17)
General well-being poor	1.08 (0.84–1.40)	1.03 (0.77–1.40)	**0.46 (0.31–0.68)**	0.77 (0.58–1.03)
Self-rated health poor or fair	1.00 (0.69–1.45)	**1.55 (1.00–2.40)**	1.06 (0.62–1.82)	0.81 (0.54–1.21)
Work-life balance poor	0.87 (0.66–1.15)	0.91 (0.68–1.23)	**0.52 (0.35–0.77)**	**0.72 (0.53–0.97)**
Strains in private life (some/a lot)	0.87 (0.67–1.13)	1.17 (0.87–1.58)	0.85 (0.59–1.24)	0.94 (0.70–1.26)
Combinations of measures				
Complete burnout syndrome (EE high, DP high and PA low) *(*Fig. 2*)*	**1.50 (1.08–2.07)**	**1.45 (1.01–2.08)**	0.66 (0.40–1.09)	0.91 (0.64–1.30)
Poor overall mental health (≥ 2 burnout symptoms, high perceived stress and poor general well-being) *(*Fig. 3a*)*	**1.65 (1.16–2.36)**	1.24 (0.83–1.83)	**0.39 (0.22–0.70)**	0.80 (0.53–1.20)
**Positive aspects**				
EE low	**1.23 (1.01–1.51)**	0.95 (0.75–1.20)	**1.56 (1.19–2.06)**	0.89 (0.76–1.12)
DP low	**0.73 (0.60–0.89)**	1.02 (0.81–1.29)	**1.77 (1.35–2.34)**	0.87 (0.69–1.09)
PA high	0.92 (0.70–1.21)	1.09 (0.80–1.50)	1.29 (0.90–1.88)	0.89 (0.66–1.20)
Job satisfaction high^b^	**0.77 (0.61–0.98)**	**0.65 (0.50–0.84)**	0.87 (0.64–1.19)	0.88 (0.67–1.15)
General well-being high	1.15 (0.94–1.41)	**1.47 (1.16–1.85)**	**2.57 (1.93–3.40)**	1.09 (0.87–1.37)
Work-life balance good	**1.31 (1.07–1.62)**	1.23 (0.97–1.57)	**2.11 (1.58–2.79)**	1.23 (0.97–1.56)
Combinations of measures				
Engagement (EE low, DP low and PA high)	1.01 (0.72–1.43)	1.26 (0.83–1.91)	**1.70 (1.07–2.70)**	0.92 (0.64–1.34)
No burnout symptoms, high general well-being and high overall job satisfaction *(*Fig. 3b*)*	0.96 (0.76–1.21)	0.93 (0.70–1.21)	1.28 (0.94–1.74)	0.86 (0.67–1.12)

^a^Low: 1st quartile (WCW-JSS score ≤ 41)

^b^High: 4th quartile (WCW-JSS score ≥ 59)

*EE* Emotional exhaustion, *DP* Depersonalisation, *PA* Personal accomplishment

**Bold** indicates statistically significant results (*p* ≤ 0.05)

Middle-aged GPs (45–59 years) more often reported burnout on all dimensions (OR = 1.45 (95% CI: 1.01–2.08), age ≤ 45 years as reference), low job satisfaction (OR = 1.44 (95% CI: 1.10–1.90)) and suboptimal self-rated health (OR = 1.55 (95% CI: 1.00–2.40)). In contrast, GPs aged ≥60 years less often reported poor overall mental health (OR = 0.39 (95% CI: 0.22–0.70)) and high perceived stress (OR = 0.49 (95% CI: 0.34–0.71)). This group also had higher likelihood of engagement (OR = 1.70 (95% CI: 1.07–2.70)), high general well-being (OR = 2.57 (95% CI: 1.93–3.40)) and good work-life balance (OR = 2.11 (95% CI: 1.58–2.79)) than their younger colleagues. GPs in group practices were significantly less likely to report poor work-life balance than GPs in solo practices (OR = 0.72 (95% CI: 0.53–0.97)).

## Discussion

### Main findings

Negative mental well-being and low job satisfaction were common among Danish GPs. One in ten met the criteria for complete burnout syndrome, one in five met the criterion for high perceived stress, and one in five reported poor general well-being. These bleak aspects overlapped, and 8% of GPs experienced poor overall mental health as a combination of poor general well-being, high stress and substantial burnout.

Still, about 25% of the GPs reported no symptoms of burnout along with high job satisfaction and general well-being. Furthermore, most GPs rated their general health as good, and only few reported to feel much burdened by strains in private life. However, the majority of GPs assessed the work-life balance as only moderate or poor.

The highest frequency of low job satisfaction and complete burnout syndrome was seen among GPs aged 45–59 years and male GPs. Male GPs also had higher risk of depersonalisation and poor overall mental health than women. Yet, female GPs more often than male GPs reported a poor work-life balance. Likewise, GPs in solo practices more often than GPs in group practices reported a poor work-life balance.

### Strengths and limitations

This nationwide survey is among the largest surveys worldwide of mental health and job satisfaction among GPs. Both the number of respondents (*N* = 1697) and the range of information retrieved are substantial. Furthermore, the unique Danish registers allowed for precise linkage of information on listed patients. Another major strength is the use of validated scales that have shown adequate internal consistency within the study population. The validity of single items constructed for the survey was investigated in a small-scale pilot test.

The identification of GPs through the PLO membership minimised the risk of sampling bias. The list was valid and adequately updated; the recorded and self-reported information on GP age and gender was consistent, and the number of GPs categorised as independent contractors within each practice was in accordance with the number reported by the GPs in 96% of the cases. However, a few of the invited GPs (*n* = 21) reported not to be active; this was mainly due to recent retirement.

The response rate was comparable to those reported in similar GP studies. In consideration of the significant size of the questionnaire, we consider this satisfactory. This study allowed for comparison of respondents and non-respondents, and response bias was present. Caring for a higher number of elderly patients, a higher number of patients with comorbidity and a more deprived patient population were all factors associated with non-response. This supports the assumption that GPs facing greater practice demands may be less likely to take out time to respond to questionnaire surveys. Thus, the proportion of GPs experiencing high levels of burnout and stress may be underestimated. The lower response rate among the oldest GPs corresponds to the findings in other GP studies [40] .

The cross-sectional design does not allow us to make any conclusions on causality. Yet, this survey provides unique opportunities for future analyses of potential causes and consequences of job satisfaction and well-being among GPs.

### Comparison with the literature

The number of Danish GPs who face complete burnout has increased twofold since 2012 and fourfold since 2004; this is mainly due to increased emotional exhaustion and depersonalisation [41, 42]. During the same period, a decrease in job satisfaction has been seen; we found a mean WCW sum score in this study at 48 compared with 55 in 2012 and 57 in 2004 [43]. This development is in accordance with the previously reported reciprocal relationship between burnout and job satisfaction among GPs [7, 44]. Earlier studies included GPs in only one region of Denmark. However, this is not expected to impair the comparability since job satisfaction and burnout did not vary significantly across regions in the current study. Compared with the general population, participating GPs had lower levels of general well-being, and more GPs perceived high levels of stress, especially in comparison with subpopulations with similar length of education [38, 45]. This is notable as we expect GPs in general to be more aware of the importance of mental health compared to professionals outside the healthcare system.

Burnout is considered a consequence of long-term job-related stress, and the emotional exhaustion component of burnout has been described as an individual stress experience. Thus, the finding that GPs more often report high emotional exhaustion than high levels of perceived stress might appear contradictory. However, the measures are intended to measure different concepts; the emotional exhaustion subscale of the MBI aims to measure job-related exhaustion (depletion of emotional resources), whereas the PSS-10 is constructed to measure perceived stress in life in general. Moreover, both scales measure symptom degree as a continuum, and the somewhat arbitrary cut-off points have been established differently. Hence, the two measures were expected to overlap, but only to some extent. Moreover, burnout and stress were expected to overlap with poor general well-being, and similar overlap between positive outcomes was also expected [27, 46]. The wide span in mental health and job satisfaction among GPs may relate to differences in self-care strategies, personality traits and other factors unrelated to work. However, differences in working conditions are likely to contribute as well. The changes in working conditions that we have witnessed in recent years may weigh heavier on GPs practicing in areas characterised by deprivation, many elderly patients and/or workforce shortage [25, 47].

The increasing prevalence of mental distress and job dissatisfaction among Danish GPs mirrors the findings from physician studies in other countries [3–6]. However, the reported prevalence of distress and dissatisfaction varies substantially across countries [10].

Few other studies have measured GP burnout using the MBI-HSS. Despite marked increases in high EE (30.6%) and high DP (21.0%) among Danish GPs, most recent GP studies from elsewhere in Europe and North America have reported even higher prevalence with high EE ranging from 46% (UK) to 53% (Ireland) and high DP ranging from 32% (Ireland) to 46% (Canada) [48–50]. Still, lower prevalence of high EE (25%) and high DP (16%) was reported in one Portuguese study [51]. Additional categorisation of burnout status based on MBI subscale scores has been approached in multiple ways, but consensus on the diagnostic criteria is lacking [26]. Only few GP studies report prevalence of GPs with high burnout scores on all MBI-HSS subscales (complete burnout syndrome). In a recent Irish study, the prevalence of GPs with complete burnout was 6.6% [48]. In earlier studies, the prevalence ranged from 3.5% in a Swiss study from 2002 to 12% (with wide inter-country variation) in a GP survey across 12 European countries from 2000 [7, 51, 52].

Low overall job satisfaction was less common in our sample (22%) than in a study among GPs in the UK from 2015 (32%) using the WCW-JSS single item [5]. In contrast, studies from Switzerland in 2009 and Norway in 2008 reported higher levels of GP job satisfaction [53, 54]. However, to our knowledge, no recent publications explore whether the job satisfaction among GPs has declined in these countries, as has been the case in Denmark.

The pronounced inter-country variation in the reported GP distress and discontentment may relate to sociodemographic and cultural differences and to different research methods and response rates [2]. However, different working conditions may also contribute substantially as the contextual factors associated with working in general practice (e.g. working hours, time per patient and type of employment) vary substantially across countries [55]. Job-related factors that are consistently reported to influence the levels of distress and dissatisfaction among GPs include lack of recognition, dissatisfaction with income, increased number of administrative tasks, long working hours, workload intensity and time pressure [13, 19]. Working conditions that undermine the GPs’ experience of providing high-quality patient care may reduce the professional fulfilment, which has been described as the satisfying inner experience of being useful and making progress and has been identified as a key motivator for physicians’ engagement in clinical work and healthcare development [56].

The finding that male GPs more often reported negative mental health and low job satisfaction is in accordance with previous research [48, 53]. Barriers to help-seeking and the propensity among physicians to ignore indicators of distress may be more pronounced in men [57]. This may contribute to increased risk of severe negative mental health among male GPs. The finding that male GPs more often reported low EE is consistent with previous research [7].

Superior mental health among GPs in the oldest age band echoes previous research [48]. This may be due to early retirement among GPs who experience low job satisfaction, workload pressure and poor health, whereas delayed retirement is seen among GPs who thrive in the job [58]. Other explanatory factors may include a cohort effect and age-related obligations unrelated to work (e.g. children living at home). This is supported by the finding that GPs of higher age reported better work-life balance. Furthermore, work-related strains may seem less intruding when retirement is approaching and you have many years of clinical experience. The high job satisfaction among the youngest GPs and the high prevalence of severe burnout symptoms among GPs in mid-career is in line with previous research [7, 52]. This increased burnout risk in middle-aged GPs compared with younger colleagues may reflect gradual development of burnout over time due to gradual depletion of resources [26].

### Implications

The high prevalence of severe mental distress implicates a need for enhanced support to the affected GPs. Special attention should be paid to subgroups at high risk (e.g. male GPs and GPs in mid-career). The variation in GP responses concerning external factors calls for future research to examine the significance of differences in size and composition of patient populations.

The substantial increase in GP distress and discontentment signifies an urgent need to address the working conditions in general practice in order to maintain a sustainable GP workforce. Further exploration of the personal and environmental factors that are related to (positive and negative) well-being and satisfaction among GPs is suggested. Initiatives aiming to enhance GP well-being, career satisfaction and engagement are recommended to strengthen primary care and support the provision of high-quality patient care in a health care sector characterised by increasing workloads and looming workforce shortage.

## Conclusion

This study documents a high and increasing level of mental distress and discontentment among GPs in Denmark. More than one in five reported low overall job satisfaction, one in three reported emotional exhaustion, and one in ten experienced complete burnout (emotional exhaustion, depersonalisation and sense of inefficacy). Furthermore, one in five perceived high levels of stress and poor general well-being. Nearly one in ten of the GPs experienced a combination of at least two burnout symptoms, high stress and poor general well-being. The risk of negative mental health and dissatisfaction was generally high for both genders and across age band and practice type, but it was particularly high among GPs in mid-career and male GPs. In contrast to the substantial minority of distressed GPs, a larger group expressed positive mental health and high job satisfaction. This indicates that GPs constitute a heterogeneous population. Targeted interventions are needed to addresses GP mental health and job satisfaction.

## Additional file

Additional file 1: Descriptive statistics of included scales, sum scores, internal consistency, and floor and ceiling effects (DOCX 15 kb)

## Abbreviations

CI: Confidence interval
DP: Depersonalisation
EE: Emotional exhaustion
GP: General practitioner
MBI-HSS: Maslach Burnout Inventory - Human Services Survey
OR: Odds ratio
PA: Personal accomplishment
PLO: Organisation of General Practitioners in Denmark
PSS-10: Cohen’s Perceived Stress Scale, 10-item Danish version
SD: Standard deviation
WCW-JSS: Warr, Cook and Wall Job Satisfaction Scale
WHO-5: The World Health Organisation Well-Being Index
