# Supplementary material for: Mental well-being and job satisfaction among general practitioners: a nationwide cross-sectional survey in Denmark
Source: BMC Fam Pract. 2018 Jul 28;19:130. doi: 10.1186/s12875-018-0809-3 (PMC6064618; doi:10.1186/s12875-018-0809-3)
Supplement: Supplementary file 1 — Descriptive statistics of included scales, sum scores, internal consistency, and floor and ceiling effects (DOCX 15 kb) [file 12875_2018_809_MOESM1_ESM.docx]

**Appendix table:** Descriptive statistics of included scales, sum scores, internal consistency, and floor and ceiling effects

|  | **General properties** | | **This survey** | | | | | | | |
| --- | --- | --- | --- | --- | --- | --- | --- | --- | --- | --- |
| **Scale (abbreviation)** | **Items** | **Scoring**  **Likert scale (range)** | **N** | **Sum score** | | | **Chronbach’s alfa** | **Average inter-item correlation** | **Floor*** (%)** | **Ceiling*** (%)** |
|  |  |  |  | **Mean (SD)** | **Min-Max** | **Median (IQI)** |  |  |  |  |
| Emotional exhaustion* (EE) | 9 | 7-point LS, (0-6) | 1.697 | 21.5 (9.3) | 0-54 | 21 (15-28) | 0.91 | 0.54 | < 0.5 | < 0.5 |
| Depersonalisation* (DP) | 5 | 7-point LS, (0-6) | 1.697 | 6.1 (4.2) | 0-25 | 6 (3-9) | 0.78 | 0.41 | 4.7 | 0.0 |
| Personal Accomplishment* (PA) | 8 | 7-point LS, (0-6) | 1.697 | 34.8 (4.8) | 10-48 | 35 (32-38) | 0.81 | 0.36 | 0.0 | < 0.5 |
| Warr Cook Wall Job Satisfaction Scale (WCW-JSS) | 10 | 7-point LS, (1-7) | 1.697 | 48.3 (13.2) | 10-70 | 51 (41-58) | 0.92 | 0.54 | 0.5 | 0.9 |
| Cohens perceived stress scale**, (PSS-10) | 10 | 5-point LS, (0-4) | 1.674 | 12.7 (6.1) | 0-33 | 13 (8-17) | 0.87 | 0.41 | 0.8 | 0.0 |
| WHO-5 well-being scale (WHO-5) | 5 | 6-point LS, (0-5) | 1.673 | 65.7 (18.2) | 0-100 | 72 (56-80) | 0.90 | 0.64 | < 0.5 | 1.6 |

*Maslach Burnout Inventory (MBI) subscale

**10-item version

***Floor/ceiling: Percentage of respondents who achieved lowest/highest possible score

LS: Likert scale, SD: standard deviation, IQI: Interquartile interval
